# Case report: Alexander's disease with “head drop” as the main symptom and literature review

**DOI:** 10.3389/fneur.2022.1002527

**Published:** 2022-12-19

**Authors:** Yujun Yuan, Qiong Wu, Liang Huo, Hua Wang, Xueyan Liu

**Affiliations:** Department of Pediatrics, Shengjing Hospital of China Medical University, Shenyang, China

**Keywords:** Alexander's disease, epilepsy, atonic seizure, GFAP, genetic disorder

## Abstract

Alexander's disease (AxD) is a rare autosomal dominant hereditary disorder that is caused by the mutations in the GFAP gene, which encodes the glial fibrillary acidic protein (GFAP). This neurogenerative disease has many clinical manifestations, and the onset of disease spans a wide range of ages, from newborns to children, adults, and even the elderly. An overaccumulation of the expression of GFAP has a close causal relationship with the pathogenesis of Alexander's disease. Usually, the disease has severe morbidity and high mortality, and can be divided into three distinct subgroups that are based on the age of clinical presentation: infantile (0–2 years), juvenile (2–13 years), and adult (>13 years). Children often present with epilepsy, macrocephaly, and psychomotor retardation, while adolescents and adults mainly present with muscle weakness, spasticity, and bulbar symptoms. Atonic seizures are a type of epilepsy that often appears in the Lennox–Gastaut syndrome and myoclonic–astatic epilepsy in early childhood; however, the prognosis is often poor. Atonic episodes are characterized by a sudden or frequent reduction in muscle tone that can be local (such as head, neck, or limb) or generalized. Here, we report a 4-year-old girl whose main symptoms were intermittent head drop movements, which could break the frontal frame and even bleed in severe conditions. A video-encephalography (VEEG) showed that the nodding movements were atonic seizures. A head magnetic resonance imaging (MRI) revealed abnormal signals in the bilateral paraventricular and bilateral subfrontal cortex. The gene detection analyses indicated that the GFAP gene exon 1 c.262 C>T was caused by a heterozygous mutation, as both her parents were of the wild-type. The girl had no other abnormal manifestations except atonic seizures. She could communicate normally and go to kindergarten. After an oral administration of sodium valproate, there were no atonic attacks. Although epilepsy is a common symptom of Alexander's disease, atonic seizures have not been reported to date. Therefore, we report a case of Alexander's disease with atonic seizures as the main symptom and provide a review of the literature.

## Introduction

W. Stewart Alexander first described Alexander's disease (AxD) in 1949 as a neurological disorder leading to leukodystrophy that primarily affects the white matter of the central nervous system (CNS). Alexander's disease is a progressive genetic disorder that primarily affects the astrocytes (1). The main pathological mechanism of Alexander's disease includes changes in GFAP gene expression that lead to the formation of Rosenthal fibers, which are composed of the glial fibrillary acidic protein (GFAP), heat shock protein 27 (HSP27), and alpha B-crystallin. Concurrently, GFAP overexpression or its abnormal degradation causes serious damage (2, 3). The proliferation of glial precursors and the dysfunction of astrocytes induce other abnormalities in the neurons and other glial cells (4). The astrocytes play an important role in the nervous system. They are involved in maintaining the homeostasis of extracellular fluids, ions, and neurotransmitters, providing energy for neurons, regulating local microcirculation, establishing and maintaining the blood–brain barrier, and maintaining the myelin sheath. The astrocytes interact with other nerves and are involved in some basic brain functions such as sleep, breathing, circadian rhythm, and memory (5, 6). Dysfunctional astrocytes are involved in hyperexcitability, neurotoxicity, and epilepsy (7). Yoshida et al. (8) found that 100% of infants, 87.5% of adolescents, and 6.3% of adults had seizures (8). The occurrence of epilepsy in an Alexander's disease is closely related to changes in the functions of astrocytes. These changes in the functions of astrocytes in Alexander's disease effect interactions between astrocytes and other CNS cells which may lead to excitotoxic damage and seizures (9). Downregulated Kir4.1 channel protein expression, altered gap junctions, and impaired glutamate uptake and metabolism have been observed in astrocytes using epileptic models (10). Epilepsy is a common clinical manifestation of Alexander's disease, and cases presenting with infantile spasms or partial status epilepticus have been reported (11, 12), yet no case with atonia as the main presentation has been reported. Atonic and tonic seizures are common in severe epilepsy during childhood, such as early infantile epileptic encephalopathy (EIEE), Lennox–Gastaut syndrome, and Doose syndrome. Furthermore, one study has found that 73% of patients presenting atonia with focal lesions had frontal lobe lesions (13).

Prust et al. (14) classified Alexander's disease as types I and II. The prognosis of type I is poor, and the main clinical manifestations of type I include epileptic seizures, encephalopathy, macrocephaly, paroxysmal deterioration, developmental disorder, developmental delay, and typical changes observed using magnetic resonance imaging (MRI) that occur in the bilateral frontal area. The main clinical manifestations of type II include brainstem symptoms, usually without neurocognitive or developmental disorders. The most common MRI findings are posterior fossa damage. However, as the symptoms develop over time, the classification of Alexander's disease also changes (14, 15).

Here, we present a case with “head drop” as the main manifestation. The brain MRI showed bilateral paraventricular–bilateral subfrontal cortex abnormal signals. Genetic testing confirmed the diagnosis of Alexander's disease. The epilepsy of the patient was well controlled after taking sodium valproate.

## Case description

**Figure 1:**
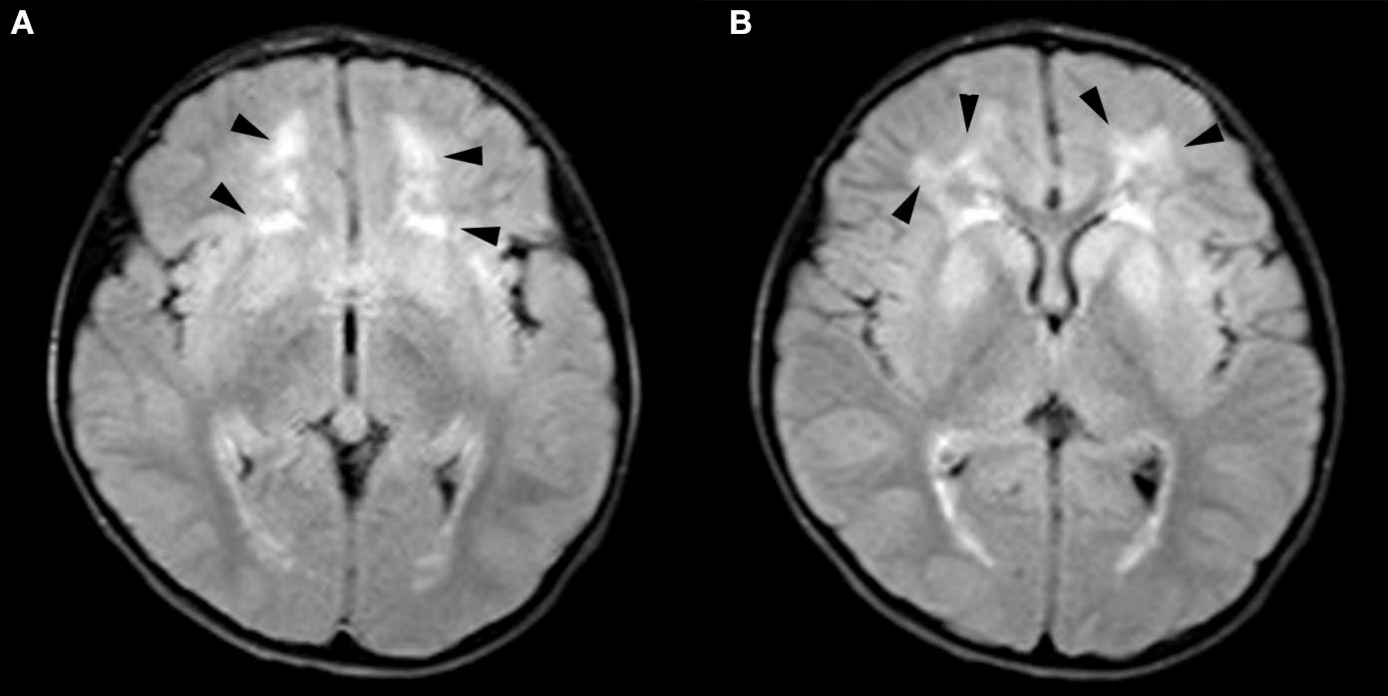
Brain magnetic resonance imaging (MRI): **(A)** Bilateral subfrontal cortex and **(B)** bilateral paraventricular abnormal signals, as displayed by the arrow.

**Figure 2:**
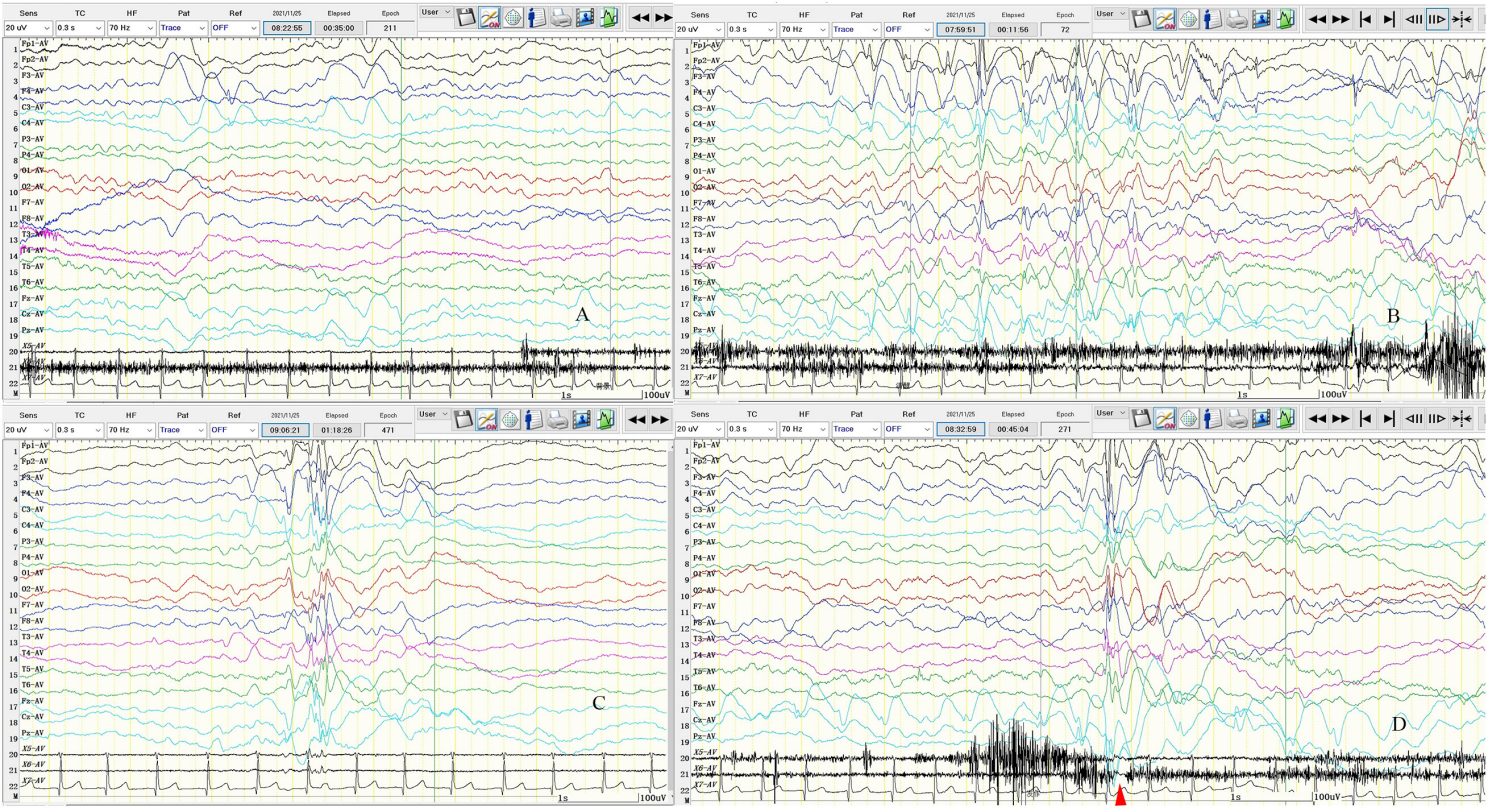
Seven-hour (7-h) video-encephalography (VEEG) on admission. **(A)** Background activity slowed down, mainly with theta (θ) wave. **(B)** The left frontal pole, frontal, central, midfrontal line, and central region of the spike-slow wave discharged. **(C)** Multiply spike-slow wave; multispike-slow wave; the slow wave is covered with spine and spikes, cluster or rhythmic discharge in both hemispheres. **(D)** Atonic seizures. The transient electrical rest appeared 20–40 ms in electromyography (EMG) after electroencephalogram (EEG) synchronous spike-slow wave burst. The clinical manifestations are head droop. X5: left upper limb; X6: left upper limb; X7: ECG; SEN: 20 uV; HF: 70; TC: 0.3; the red arrow is the electric rest.

**Figure 3:**
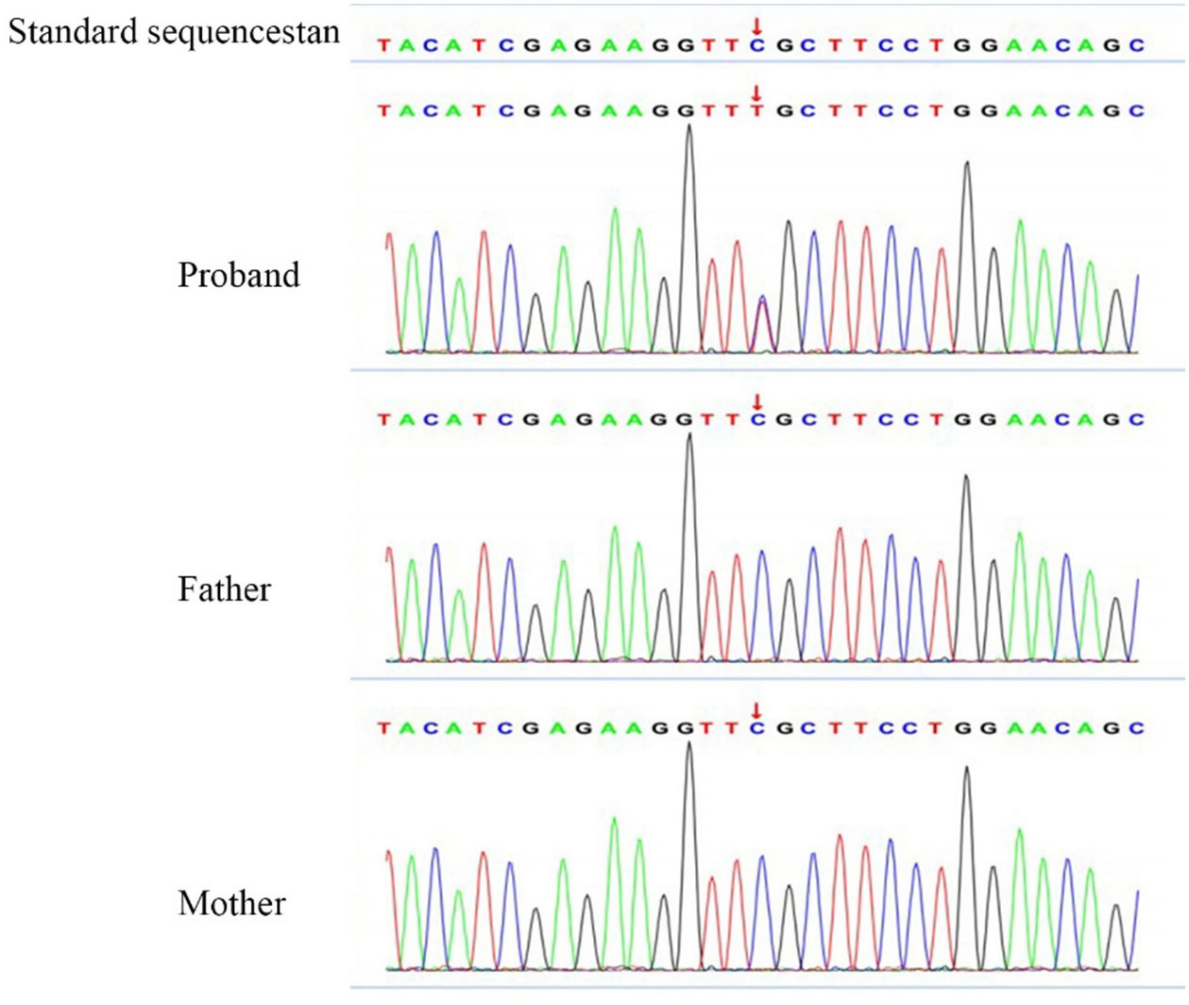
Gene testing results. Location of the chromosome: Chr17:42992593. Gene mutation in c.262 (exon1) C>T was observed, due to the 88th amino acid change from arginine to cysteine. RS: 61622935. No variant was found in the parent.

A 4-year-old girl was admitted to our department (Shengjing Hospital affiliated to China Medical University, Department of Pediatric Neurology, Shenyang, China) with an “intermittent head drop” for 5 months as the chief complaint. The main clinical manifestations were: a sudden head downward movement one to three times a day and often after waking up in the morning. Two weeks before admission, the above symptom aggravated to 5–6 times in a day. The patient had a history of convulsions at the age of 1 year and seven months. The clinical manifestations were a sudden loss of consciousness, being unresponsive after calling, eyes turning up, limbs collapsing, and no cyanosis. The episode lasted ~10 min without fever. The child was the second born of a non-consanguineous marriage with a normal birth history. She exhibited normal physical growth and neurologic developmental milestones. The head circumference measured at birth was 33 cm (15th percentile), the height was 51 cm (50th percentile), and the weight was 3.025 kg (50th percentile). Now (5 years old), the head circumference measured is 49 cm (15th percentile), the height is 112 cm (50th percentile), and the weight is 20.5 kg (75th percentile). The examination of the nervous system after admission showed clear consciousness, fluent answers, sensitive pupils to light reflex, no nystagmus, tongue in the center, no tongue muscle tremor, limb muscle tone normal, muscle strength grade 5, motor coordination, neck strength (–), and Babinski sign (–). Furthermore, the finger–nose, rotational exercise, heel–knee–tibial, and Romberg tests for imbalance were normal. No abnormal positive neurological signs were observed. The biochemical examination showed no significant abnormality, and the brain MRI+DWI (diffusion-weighted imaging) showed bilateral paraventricular–bilateral subfrontal cortex abnormal signals (Figure 1). A seven-hour video-encephalography (VEEG) after admission (Figure 2) showed a slow background, numerous abnormal epileptiform discharges during the awake and sleep stages, slow waves that increased, and atonic seizures that were monitored twice. The clinical manifestation of atonic seizures was the head drop movement. Therefore, the girl was administered 5 ml of sodium valproate orally twice daily. After 3 days, the child had no seizures and was discharged. Genetic testing was performed during the hospital stay (at the age of 4), and 20 days later, the genetic testing results showed that GFAP gene exon 1 c.262 C>T, and that the pathogenic mutations could lead to the 88th amino acid change from arginine to cysteine (Figure 3). The patients presented a heterozygous mutation; both parents were of the wild-type, with no history of convulsions or a definite disease. The sister of the patient is currently healthy and attending primary school. The patient was eventually diagnosed with Alexander's disease. A classification as type I or type II at present is not possible because the only symptom is epilepsy (unlike type I), and no brainstem symptoms or motor abnormalities (unlike type II) were observed. If clinical symptoms emerge over time, a better classification may be possible.

**Figure 4:**
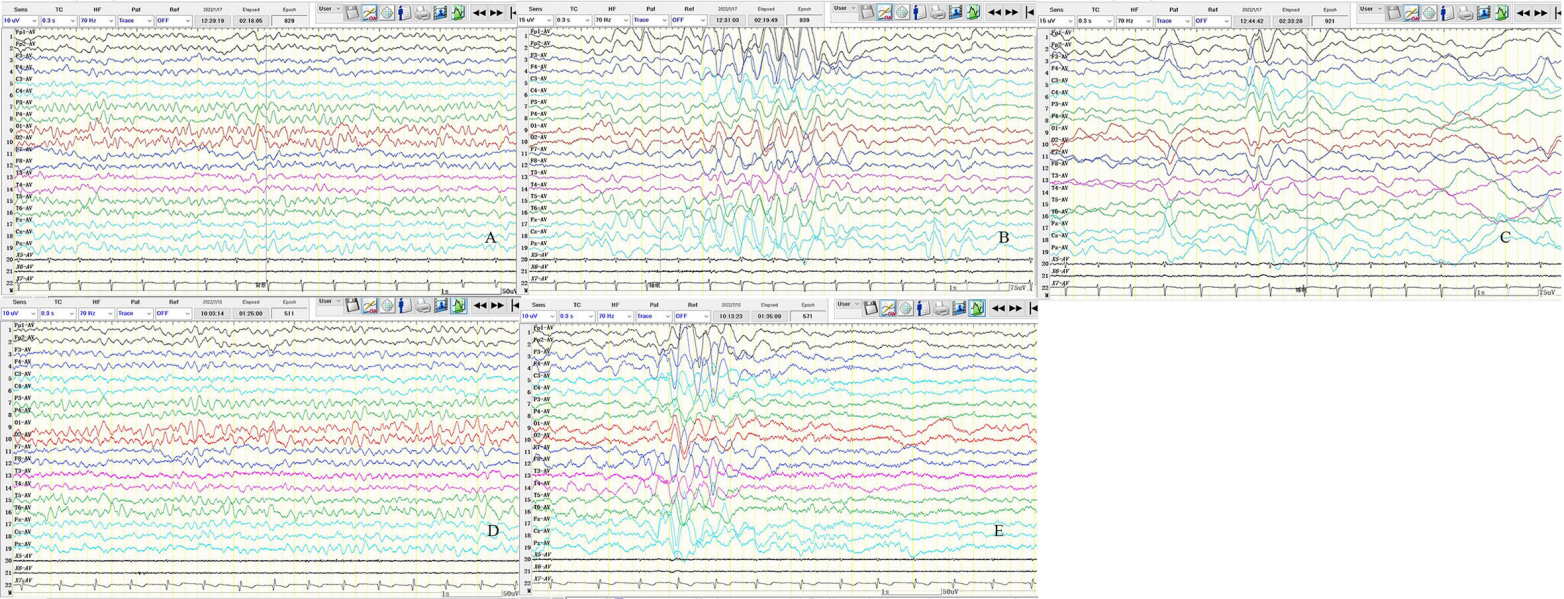
Results of two video-encephalographies (VEEGs) performed 35 days after discharge **(A–C)** and 7 months after discharge **(D,E)**. **(A)** The 8–9 Hz medium amplitude α wave rhythm in the bilateral occipital area. **(B)** The 3–4 Hz slow waves in the bilateral anterior head are asynchronous or rhythmic discharge. **(C)** Slow wave or sharp slow wave discharged in the bilateral frontal, central, parietal, and midline areas asynchronously. **(D)** The 6–7 Hz medium amplitude θ wave rhythm in the bilateral occipital area. **(E)** The 3–4 Hz slow waves discharge in sleep, and the bilateral anterior head is predominant. X5, Left upper limb; X6, left upper limb; X7, ECG; SEN,10 uV in A, D, E and 15 uV in B, C; HF, 70; TC, 0.3.

We asked the patient to perform regular liver and kidney function tests, undergo blood routine examination, and monitor the blood drug concentration of sodium valproate, so as to adjust the antiepileptic medication based on professional indications. The family was asked to watch for other seizures and behavior and any other cognitive changes. After discharge, the patient had two VEEGs, one was performed 53 days after discharge, which showed: (1) Asynchronous or rhythmic discharge of 3–4 Hz slow waves in the bilateral anterior head. (2) Asynchronously slow wave or sharp slow wave discharged in the bilateral frontal, central, parietal, and midline areas. The second VEEG was performed seven months after discharge (at the age of 5)and only showed a few 3 Hz slow waves during sleep, yet the background was a bit slow (Figure 4). No convulsions were recorded since taking sodium valproate, and no adverse reactions occurred during the treatment. The patient communicates normally. To date, the patient has presented no obvious abnormality or backwardness in psychomotor and cognition.

## Discussion

Alexander's disease, a rare autosomal dominant leukodystrophy disorder, is characterized by a progressive central demyelination injury, Rosenthal fibrous accumulation, and leukodystrophy changes due to shifts in GFAP caused by the mutations in GFAP gene (16). GFAP is an intermediate silk protein that emerged early during vertebrate evolution (17), and the inner filament is an important component of the cytoplasm cytoskeleton that performs many diverse functions such as a structural support, a scaffold for enzymes and organelles, and also to help in mechanical sensing in the extracellular environment (18). GFAP is generally expressed at low levels in the cerebrospinal fluid (CSF) and blood, but when the central nervous system is damaged or affected by any disease, the level of GFAP increases accordingly, especially after traumatic events, stroke, subarachnoid hemorrhage, or neuromyelitis optica (19, 20). Therefore, since the time it was discovered, GFAP has been used as a biomarker for astrocytes (21).

The glial fibrillary acidic protein has a central rod domain and a head (N-terminal) and a tail (C-terminal) domain. The central rod domain consists of four non-helical domains(1A, 1B, 2A, and 2B). The intermediate filaments and domains 1A and 2B make a difference in forming dimers, tetramers, and protofibrils in the GFAP (22). Different variants of GFAP lead to different forms of Alexander's disease. Most GFAP mutations reported are *de novo* mutations with a penetrance close to 100% (23, 24). Approximately 290 AxD-related mutations result in ~100 different amino acid changes, most of which are point mutations. However, there are other forms of mutations, such as insertion and deletion. A homozygous mutation of the GFAP gene was reported in Taiwan (25). Missense mutations can occur anywhere in the GFAP structure, but it is more common to find these mutations in the rod-shaped and tail domains. An analysis of the 215 patients diagnosed with Alexander's disease showed that the incidence rate in exons 1, 3, 4, 5, 6, 7, and 8 is 45.5%, 3.3%, 27.2%, 1.8%, 16%, <1%, and 7.5%, respectively, with coli1A as the most common domain (14). The hottest mutation spots are p.R79, p.R88, p.R239, and p.R416. R79 and R239 were the most common early-onset types of AxD; however, patients with R88 and R416 did not present a significant genotype–phenotype correlation (14, 23). Astrocytes differentiated from patients carrying R88C by displaying changes in intracellular vesicle transport, calcium dynamics, and adenosine triphosphate (ATP) release (26). Our patient had a mutation at R88 of GFAP, a pathogenic mutation that could lead to the 88th amino acid change from arginine to cysteine. However, the same gene mutation could lead to different clinical manifestations, including the early- and late-onset AxD caused by the R88C mutation (23, 27). Zardadi et al. (28) reported a patient with R88 whose main clinical presentation was area postrema-like syndrome, and the symptoms improved with administration of prednisolone. This patient also had generalized atonic seizures in the early stages. Furthermore, a patient with an R88C mutation with microcephaly was reported in India. Several novel variants have recently been reported, including c.778 A > C in exon 4 (29); c1289G>A, c.1290C >A (30); c.726_728dupAGG in exon 4 (31); and c.382 G>A (32).

A common symptom of Alexander's disease is epilepsy, especially in type I AxD. Pathogenesis of epilepsy in Alexander's disease is very complex, and at present, there is no clear explanation to offer on the complexity of epileptic symptoms in a patient. However, epilepsy may be caused by the astrocyte extracellular ion concentration, neurotransmitter changes (especially glutamate), energy metabolism, activation of stress responses, and functional impairment of the gap junction in an astrocyte network (33). The reports of epilepsy in Alexander's disease include tonic–clonic seizures (27), infantile spasms (11), and persistent epilepsy (12, 34). However, the case of atonic seizures as the main manifestation has not been reported. In the International League Against Epilepsy (ILAE) classification, “atonic seizures” are described as sudden reductions in muscle tone, which can be sporadic with drooping of the head, jaw, and limbs or even falling to the ground due to the loss of muscle tone (35). The loss of tension takes place in a local muscle group or is generalized. Patients often suffer severe trauma due to episodes of atonia (36, 37), which are often associated with early childhood severe epileptic encephalopathy or epilepsy syndromes, such as myoclonic–atonic seizures and Lennox–Gastaut syndrome. Besides, they are often associated with other types of seizures, such as atypical absence, myoclonic seizures, complex partial seizures, tonic seizures (38, 39), and acute epileptic encephalopathy changes. In addition to frequent seizures, children show decreased alertness, reduced social interaction, lethargy, language difficulties, and ataxia (40, 41). Sodium valproate is the first choice for treating various comprehensive and undefined types of epilepsy. At present, there is no first-line drug for atonic seizures. Alternative drugs include sodium valproate, lamotrigine, and topiramate (42, 43). A ketogenic diet also has a good controlling effect on drop attacks (44). In recent years, some new drugs, such as clobazam and cannabidiol (CBD), have been tried to treat refractory epilepsy. Highly purified CBD has been approved for the treatment of seizures associated with Dravet syndrome, Lennox–Gastaut syndrome, and tuberous sclerosis complex (45). Studies have shown that patients with Doose syndrome had a 58.6% reduction in seizure frequency at week 12 after the administration of high-purity cannabidiol (45, 46). Clobazam can be used as an add-on therapy for treatment of atonic epilepsy or some refractory epilepsy (47–49). It was even recommended as the first-line therapy for treatment of myoclonic–atonic seizures (50). In addition to medical treatment, some surgical treatments significantly improve refractory atonic seizures, such as corpus callosotomy (CC) and vagus nerve stimulation (VNS), both CC and VNS being well tolerated for the treatment of refractory atonic seizures (50–52).

Episodes of atonia and tonus are associated with frontal lobes containing primary motor cortices and negative motor areas. Baraldi et al. (13) found focal lesions in 39% of patients with falls, 73% of which were frontal lobe lesions, and the presumed epileptic region was also usually located in the frontal lobe (43%). Stimulating the cerebral cortex can trigger a response from the corresponding muscles. The motor regions associated with inhibitory responses are widely distributed in the lateral and mesial frontal cortex, known as primary or auxiliary negative motor regions (53). Based on the movement disorder seizures outlined by Noachtar and Luders (35), atonic seizures are a subset of akinetic epilepsy because there are no positive seizure-like seizures other than the loss of tension. In 2006, Rubboli et al. (54) described clinically insignificant twitching or loss of muscle tone in the opposite deltoid when stimulating the supplementary motor area with a 1 Hz single electric pulse. Similarly, Ikeda et al. (55) also found that contralateral inhibitory motor responses would occur when a part of the primary sensorimotor cortex was stimulated. Electroclinical studies also demonstrated that the epileptogenic region of atonic seizures is located in the premotor region, rostral, or adjacent primary motor cortex (56). In the case presented here, the patient also had a frontal lobe lesion, and VEEG showed a significant abnormal discharge in the frontal region.

Atonic epilepsy should be differentiated from Todd's palsy due to focal epilepsy (57). However, atonia and negative myoclonus can have the same symptoms, but the latter has been considered a temporary atonic seizure. Since fall attacks can be positive manifestations of atonic and tonic seizures, it is difficult to distinguish atonic seizures from tonic seizures without the aid of EEG and EMG (58, 59). Atonic seizures are also distinguished from akinetic seizures, characterized by a sudden stop of speech and movement and inability to move, speak, or follow commands, even when the patient remains conscious and wake. After the seizure, patients can recall what others had asked them during the seizure and describe their state during the seizure. When the seizure ends, they return to their previous activities without language or cognitive changes (60, 61).

Diagnosing Alexander's disease is confirmed by genetic testing; however, some patients cannot achieve a perfect genetic testing. Clinicians can assist in the diagnosis by its specific clinical manifestations and specific MRI changes. MRI criteria for diagnosing Alexander's disease were drafted in 2001 and included five main areas (62). Alexander's disease has many atypical variations in brain MRI, which may not completely meet the standard of typical MRI, even when changes are observed. This variation or atypia is more common in patients with a later onset and slower course of the disease (63). Special changes in MRI must be identified with other genetic metabolic diseases, such as Adrenoleukodystrophy, Canavan's disease, Metachromatic leukodystrophy, Pelizaeus–Merzbacher disease, and Tay–Sachs disease (1, 12).

The GFAP overexpression plays an important role in the pathogenesis of AxD. Intracellular GFAP mutations of up to 20% can trigger the disease (64). Thus, inhibiting the expression of GFAP helps to alleviate the disease. Bachetti et al. (65) demonstrated that ceftriaxone can reduce the intracytoplasmal aggregation of mutated GFAP in Alexander's disease cell models, eliminate the expression of mutated GFAP, regulate the proteasome system, reduce the activation of NF-kappa B (NF-kB), and downregulate the expression of GFAP levels. Meanwhile, Sechi et al. (66) reported that ataxia and dysarthria in an adult patient were relieved after cephalosporin was administered. Hagemann et al. (4) reported that antisense oligonucleotides (ASOs) serve as an excellent source to suppress GFAP expression. He demonstrated that injecting antisense oligonucleotides (ASOs) with the help of single bolus intracerebroventricular injections into a mouse model showed significant inhibitory effects on GFAP expression. Changing the entire course of Alexander's disease, they showed that almost all GFAP and Rosenthal fibers were eliminated after treatment of the Alexander mouse model. In addition, the antidepressant clomipramine can inhibit the GFAP expression *in vitro* and using animal models (67, 68). However, long-term use of these drugs can increase the risk of seizures, especially if there is a history of nerve damage (69), decreasing the potential widespread use of the drug in patients, especially in type I AxD. Bachetti et al. (70) demonstrated for the first time that carbamazepine and phenytoin could inhibit the expression and folding of pathological GFAP cells, thus leading to the reduced formation of mutant GFAP aggregates. Therefore, carbamazepine and phenytoin sodium may have potential therapeutic effects on AxD, especially in patients with partial epilepsy of AxD. Combined with our case, the epilepsy type was atonia, which is a generalized seizure and there are numerous generalized and focal discharges during the interictal phase. According to our clinical experience, we prescribed the oral delivery of the broad-spectrum antiepileptic sodium valproate drug. The patient's symptoms were significantly relieved after the medication, and VEEG significantly improved.

## Conclusion

Epilepsy is a common symptom of Alexander's disease; however, it is rare to come across patients presenting with atonic seizures as the main symptoms. At this point of time, it is absolutely necessary to enhance the spread of awareness about Alexander's disease, especially epilepsy, and representative head MRI changes in the same patient at the same time should also consider some special diseases, such as hereditary, metabolic, or rare diseases. In this study, genetic testing has played a phenomenal role by confirming Alexander's disease and it has now become important to help us in diagnosing diseases. Additionally, there is no fundamental effective drug treatment for Alexander's disease, which warrants our attention.

## Data availability statement

The datasets presented in this article are not readily available because of ethical and privacy restrictions. Requests to access the datasets should be directed to the corresponding author/s.

## Ethics statement

Written informed consent was obtained from the minor(s)' legal guardian/next of kin for the publication of any potentially identifiable images or data included in this article.

## Author contributions

YY: collected the case and drafted the article. QW and LH: collected and analyzed the case. XL and HW: designed and guided the work and revised the manuscript. All authors contributed to the article and approved the submitted version.

## References

[1] Kuhn J Cascella M. Alexander disease. In: StatPearls [Internet]. Treasure Island, FL: StatPearls Publishing (2022). 32965913

[2] Lin NH Huang YS Opal P Goldman RD Messing A Perng MD. The role of gigaxonin in the degradation of the glial-specific intermediate filament protein GFAP. Mol Biol Cell. (2016) 27:3980–90. 10.1091/mbc.E16-06-0362 27798231 PMC5156539

[3] Hagemann TL Connor JX Messing A. Alexander disease-associated glial fibrillary acidic protein mutations in mice induce Rosenthal fiber formation and a white matter stress response. J Neurosci. (2006) 26:11162–73. 10.1523/JNEUROSCI.3260-06.2006 17065456 PMC6674663

[4] Hagemann TL Powers B Mazur C Kim A Wheeler S Hung G. Antisense suppression of glial fibrillary acidic protein as a treatment for Alexander disease. Ann Neurol. (2018) 83:27–39. 10.1002/ana.25118 29226998 PMC5876100

[5] Pavlou MAS Grandbarbe L Buckley NJ Niclou SP Michelucci A. Transcriptional and epigenetic mechanisms underlying astrocyte identity. Prog Neurobiol. (2019) 174:36–52. 10.1016/j.pneurobio.2018.12.007 30599178

[6] Sofroniew MV. Astrocyte Reactivity: Subtypes, States, and Functions in CNS Innate Immunity. Trends Immunol. (2020) 41:758–70. 10.1016/j.it.2020.07.004 32819810 PMC7484257

[7] Sanz P Garcia-Gimeno MA. Reactive Glia Inflammatory Signaling Pathways and Epilepsy. Int J Mol Sci. (2020) 21:4096. 10.3390/ijms21114096 32521797 PMC7312833

[8] Yoshida T Sasaki M Yoshida M Namekawa M Okamoto Y Tsujino S. Nationwide survey of Alexander disease in Japan and proposed new guidelines for diagnosis. J Neurol. (2011) 258:1998–2008. 10.1007/s00415-011-6056-3 21533827

[9] Sosunov AA Guilfoyle E Wu X 2nd GMM Goldman JE. Phenotypic conversions of “protoplasmic” to “reactive” astrocytes in Alexander disease. J Neurosci. (2013) 33:7439–50. 10.1523/JNEUROSCI.4506-12.2013 23616550 PMC3694721

[10] Coulter DA Steinhauser C. Role of astrocytes in epilepsy. Cold Spring Harb Perspect Med. (2015) 5:a22434. 10.1101/cshperspect.a022434 25732035 PMC4355248

[11] Lee JM Kim AS Lee SJ Cho SM Lee DS Choi SM. A case of infantile Alexander disease accompanied by infantile spasms diagnosed by DNA analysis. J Kor Med Sci. (2006) 21:954–7. 10.3346/jkms.2006.21.5.954 17043438 PMC2722014

[12] Bonthius DJ Karacay B. Alexander disease: a novel mutation in GFAP leading to *Epilepsia partialis* continua. J Child Neurol. (2016) 31:869–72. 10.1177/0883073815624762 26719496 PMC4865433

[13] Baraldi S Farrell F Benson J Diehl B Wehner T Kovac S. Drop attacks, falls and atonic seizures in the Video-EEG monitoring unit. Seizure. (2015) 32:4–8. 10.1016/j.seizure.2015.08.001 26552554

[14] Prust M Wang J Morizono H Messing A Brenner M Gordon E. GFAP mutations, age at onset, and clinical subtypes in Alexander disease. Neurology. (2011) 77:1287–94. 10.1212/WNL.0b013e3182309f72 21917775 PMC3179649

[15] Mura E Nicita F Masnada S Battini R Ticci C Montomoli M. Alexander disease evolution over time: data from an Italian cohort of pediatric-onset patients. Mol Genet Metab. (2021) 134:353–8. 10.1016/j.ymgme.2021.11.009 34865968

[16] Sawaishi Y. Review of Alexander disease: beyond the classical concept of leukodystrophy. Brain Dev. (2009) 31:493–8. 10.1016/j.braindev.2009.03.006 19386454

[17] Wicht H Derouiche A Korf HW. An immunocytochemical investigation of glial morphology in the Pacific hagfish: radial and astrocyte-like glia have the same phylogenetic age. J Neurocytol. (1994) 23:565–76. 10.1007/BF01262057 7815088

[18] Lowery J Kuczmarski ER Herrmann H Goldman RD. Intermediate filaments play a pivotal role in regulating cell architecture and function. J Biol Chem. (2015) 290:17145–53. 10.1074/jbc.R115.640359 25957409 PMC4498054

[19] Liem RK Messing A. Dysfunctions of neuronal and glial intermediate filaments in disease. J Clin Invest. (2009) 119:1814–24. 10.1172/JCI38003 19587456 PMC2701870

[20] Petzold A. Glial fibrillary acidic protein is a body fluid biomarker for glial pathology in human disease. Brain Res. (2015) 1600:17–31. 10.1016/j.brainres.2014.12.027 25543069

[21] Messing A Brenner M GFAP. at 50. ASN Neuro. (2020) 12:1665526960. 10.1177/1759091420949680 32811163 PMC7440737

[22] Heshmatzad K Panah MH Tavasoli AR Ashrafi MR Mahdieh N Rabbani B. GFAP variants leading to infantile Alexander disease: phenotype and genotype analysis of 135 cases and report of a de novo variant. Clin Neurol Neurosurg. (2021) 207:106754. 10.1016/j.clineuro.2021.106754 34146839

[23] Brenner M Johnson AB Boespflug-Tanguy O Rodriguez D Goldman JE Messing A. Mutations in GFAP, encoding glial fibrillary acidic protein, are associated with Alexander disease. Nat Genet. (2001) 27:117–20. 10.1038/83679 11138011

[24] Rodriguez D Gauthier F Bertini E Bugiani M Brenner M N'guyen S. Infantile Alexander disease: spectrum of GFAP mutations and genotype-phenotype correlation. Am J Hum Genet. (2001) 69:1134–40. 10.1086/323799 11567214 PMC1274357

[25] Fu MH Chang YY Lin NH Yang AW Chang CC Liu JS. Recessively-inherited adult-onset alexander disease caused by a homozygous mutation in the GFAP Gene. Mov Disord. (2020) 35:1662–7. 10.1002/mds.28099 32374915

[26] Lanciotti A Brignone MS Macioce P Visentin S Ambrosini E. Human iPSC-derived astrocytes: a powerful tool to study primary astrocyte dysfunction in the pathogenesis of rare leukodystrophies. Int J Mol Sci. (2021) 23:274. 10.3390/ijms23010274 35008700 PMC8745131

[27] Wu Y Gu Q Wang J Yang Y Wu X Jiang Y. Clinical and genetic study in Chinese patients with Alexander disease. J Child Neurol. (2008) 23:173–7. 10.1177/0883073807308691 18079314

[28] Zardadi S Razmara E Rasoulinezhad M Babaei M Ashrafi MR Pak N. Symptomatic care of late-onset Alexander disease presenting with area postrema-like syndrome with prednisolone; a case report. BMC Pediatr. (2022) 22:412. 10.1186/s12887-022-03468-y 35831840 PMC9277918

[29] Zaver DB Douthit NT. A Novel Mutation in the Adult-Onset Alexander's Disease GFAP Gene. Case Rep Med. (2019) 2019:2986538. 10.1155/2019/2986538 30755773 PMC6348877

[30] Helman G Takanohashi A Hagemann TL Perng MD Walkiewicz M Woidill S. Type II Alexander disease caused by splicing errors and aberrant overexpression of an uncharacterized GFAP isoform. Hum Mutat. (2020) 41:1131–7. 10.1002/humu.24008 32126152 PMC7491703

[31] Yasuda R Yoshida T Mizuta I Nakagawa M Mizuno T. A novel three-base duplication, E243dup, of GFAP identified in a patient with Alexander disease. Hum Genome Var. (2017) 4:17028. 10.1038/hgv.2017.28 28690862 PMC5498426

[32] Chang K-E Pratt D Mishra BB Edwards N Hallett M Ray-Chaudhury A. Type II (adult onset) Alexander disease in a paraplegic male with a rare D128N mutation in the GFAP gene. Clin Neuropathol. (2015) 34:298–302. 10.5414/NP300863 25997626 PMC4766795

[33] Boison D Steinhauser C. Epilepsy and astrocyte energy metabolism. Glia. (2018) 66:1235–43. 10.1002/glia.23247 29044647 PMC5903956

[34] Nair RR. Alexander's disease presenting as status epilepticus in a child. J Postgrad Med. (2005) 51:244. Available online at: https://www.jpgmonline.com/text.asp?2005/51/3/244/19038 16333210

[35] Kovac S Diehl B. Atonic phenomena in focal seizures: nomenclature, clinical findings and pathophysiological concepts. Seizure. (2012) 21:561–7. 10.1016/j.seizure.2012.06.004 22789404

[36] Proposal for revised clinical and electroencephalographic classification of epileptic seizures. From the commission on classification and terminology of the international league against epilepsy. Epilepsia. (1981) 22:489–501. 10.1111/j.1528-1157.1981.tb06159.x 6790275

[37] Villani F D'Amico D Pincherle A Tullo V Chiapparini L Bussone G. Prolonged focal negative motor seizures: a video-EEG study. Epilepsia. (2006) 47:1949–52. 10.1111/j.1528-1167.2006.00804.x 17116038

[38] Dragoumi P Chivers F Brady M Craft S Mushati D Venkatachalam G. Epilepsy with myoclonic-atonic seizures (Doose syndrome): When video-EEG polygraphy holds the key to syndrome diagnosis. Epilepsy Behav Case Rep. (2016) 5:31–3. 10.1016/j.ebcr.2015.10.001 26958468 PMC4773482

[39] Devinsky O Patel AD Cross JH Villanueva V Wirrell EC Privitera M. Effect of Cannabidiol on Drop Seizures in the Lennox-Gastaut Syndrome. N Engl J Med. (2018) 378:1888–97. 10.1056/NEJMoa1714631 29768152

[40] Kaminska A Oguni H. Lennox-Gastaut syndrome and epilepsy with myoclonic-astatic seizures. Handb Clin Neurol. (2013) 111:641–52. 10.1016/B978-0-444-52891-9.00067-1 23622212

[41] Kaminska A Ickowicz A Plouin P Bru MF Dellatolas G Dulac O. Delineation of cryptogenic Lennox-Gastaut syndrome and myoclonic astatic epilepsy using multiple correspondence analysis. Epilepsy Res. (1999) 36:15–29. 10.1016/S0920-1211(99)00021-2 10463847

[42] Hakami T. Neuropharmacology of Antiseizure Drugs. Neuropsychopharmacol Rep. (2021) 41:336–51. 10.1002/npr2.12196 34296824 PMC8411307

[43] Liu G Slater N Perkins A. Epilepsy: treatment options. Am Fam Physician. (2017) 96:87–96. Available online at: https://www.aafp.org/pubs/afp/issues/2017/0715/p87.html 28762701

[44] Vining EP. Tonic and atonic seizures: medical therapy and ketogenic diet. Epilepsia. (2009) 50 Suppl 8:21–4. 10.1111/j.1528-1167.2009.02231.x 19702729

[45] Lattanzi S Trinka E Striano P Rocchi C Salvemini S Silvestrini M. Highly purified cannabidiol for epilepsy treatment: a systematic review of epileptic conditions beyond dravet syndrome and lennox-gastaut syndrome. CNS Drugs. (2021) 35:265–81. 10.1007/s40263-021-00807-y 33754312 PMC8005394

[46] Lattanzi S Trinka E Striano P Rocchi C Salvemini S Silvestrini M. Open-label use of highly purified CBD (Epidiolex(R)) in patients with CDKL5 deficiency disorder and Aicardi, Dup15q, and Doose syndromes. Epilepsy Behav. (2018) 86:131–7. 10.1016/j.yebeh.2018.05.013 30006259

[47] Trivisano M Striano P Sartorelli J Giordano L Traverso M Accorsi P. CHD2 mutations are a rare cause of generalized epilepsy with myoclonic-atonic seizures. Epilepsy Behav. (2015) 51:53–6. 10.1016/j.yebeh.2015.06.029 26262932

[48] Vlaskamp DRM Rump P Callenbach PMC Vos YJ Sikkema-Raddatz B van Ravenswaaij-Arts CMA. Haploinsufficiency of the STX1B gene is associated with myoclonic astatic epilepsy. Eur J Paediatr Neurol. (2016) 20:489–92. 10.1016/j.ejpn.2015.12.014 26818399

[49] Kalra V Seth R Mishra D Saha NC. Clobazam in refractory childhood epilepsy. Indian J Pediatr. (2010) 77:263–6. 10.1007/s12098-010-0035-z 20177827

[50] Joshi C Nickels K Demarest S Eltze C Cross JH Wirrell E. Results of an international Delphi consensus in epilepsy with myoclonic atonic seizures/ Doose syndrome. Seizure. (2021) 85:12–8. 10.1016/j.seizure.2020.11.017 33383403

[51] Ye VC Mansouri A Warsi NM Ibrahim GM. Atonic seizures in children: a meta-analysis comparing corpus callosotomy to vagus nerve stimulation. Childs Nerv Syst. (2021) 37:259–67. 10.1007/s00381-020-04698-0 32529546

[52] Rolston JD Englot DJ Wang DD Garcia PA Chang EF. Corpus callosotomy versus vagus nerve stimulation for atonic seizures and drop attacks: a systematic review. Epilepsy Behav. (2015) 51:13–7. 10.1016/j.yebeh.2015.06.001 26247311 PMC5261864

[53] Luders HO Dinner DS Morris HH. Cortical electrical stimulation in humans. The negative motor areas. Adv Neurol. (1995) 67:115–29. 8848964

[54] Rubboli G Mai R Meletti S Francione S Cardinale F Tassi L. Negative myoclonus induced by cortical electrical stimulation in epileptic patients. Brain. (2006) 129:65–81. 10.1093/brain/awh661 16272166

[55] Ikeda A Ohara S Matsumoto R Kunieda T Nagamine T Miyamoto S. Role of primary sensorimotor cortices in generating inhibitory motor response in humans. Brain. (2000) 123:1710–21. 10.1093/brain/123.8.1710 10908200

[56] Scholly J Bartolomei F Valenti-Hirsch MP Boulay C Martin AD Timofeev A. Atonic seizures in children with surgically remediable epilepsy: a motor system seizure phenotype? Epileptic Disord. (2017) 19:315–26. 10.1684/epd.2017.0930 28832003

[57] Gallmetzer P Leutmezer F Serles W Assem-Hilger E Spatt J Baumgartner C. Postictal paresis in focal epilepsies–incidence, duration, and causes: a video-EEG monitoring study. Neurology. (2004) 62:2160–4. 10.1212/WNL.62.12.2160 15210875

[58] Egli M Mothersill I O'Kane M O'Kane F. The axial spasm–the predominant type of drop seizure in patients with secondary generalized epilepsy. Epilepsia. (1985) 26:401–15. 10.1111/j.1528-1157.1985.tb05671.x 4043010

[59] Ikeno T Shigematsu H Miyakoshi M Ohba A Yagi K Seino M. An analytic study of epileptic falls. Epilepsia. (1985) 26:612–21. 10.1111/j.1528-1157.1985.tb05701.x 3935426

[60] Lüders H Acharya J Baumgartner C Benbadis S Bleasel A Burgess R. Semiological seizure classification. Epilepsia. (1998) 39:1006–13. 10.1111/j.1528-1157.1998.tb01452.x 9738682

[61] Toledano R García-Morales I Kurtis MM Pérez-Sempere A Ciordia R Gil-Nagel A. Bilateral akinetic seizures: a clinical and electroencephalographic description. Epilepsia. (2010) 51:2108–15. 10.1111/j.1528-1167.2010.02662.x 20662894

[62] van der Knaap MS Naidu S Breiter SN Blaser S Stroink H Springer S. Alexander disease: diagnosis with MR imaging. AJNR Am J Neuroradiol. (2001) 22:541–52. 11237983 PMC7976831

[63] van der Knaap MS Salomons GS Li R Franzoni E Gutiérrez-Solana LG Smit LME. Unusual variants of Alexander's disease. Ann Neurol. (2005) 57:327–38. 10.1002/ana.20381 15732098

[64] Hagemann TL Gaeta SA Smith MA Johnson DA Johnson JA Messing A. Gene expression analysis in mice with elevated glial fibrillary acidic protein and Rosenthal fibers reveals a stress response followed by glial activation and neuronal dysfunction. Hum Mol Genet. (2005) 14:2443–58. 10.1093/hmg/ddi248 16014634

[65] Bachetti T Di Zanni E Balbi P Bocca P Prigione I Deiana GA. *In vitro* treatments with ceftriaxone promote elimination of mutant glial fibrillary acidic protein and transcription down-regulation. Exp Cell Res. (2010) 316:2152–65. 10.1016/j.yexcr.2010.05.005 20471977

[66] Sechi G Ceccherini I Bachetti T Deiana GA Sechi E Balbi P. Ceftriaxone for Alexander's disease: a four-year follow-up. JIMD Rep. (2013) 9:67–71. 10.1007/8904_2012_180 23430549 PMC3565626

[67] Cho W Brenner M Peters N Messing A. Drug screening to identify suppressors of GFAP expression. Hum Mol Genet. (2010) 19:3169–78. 10.1093/hmg/ddq227 20538881 PMC2908470

[68] Liu D Wang Z Gao Z Xie K Zhang Q Jiang H. Effects of curcumin on learning and memory deficits, BDNF, and ERK protein expression in rats exposed to chronic unpredictable stress. Behav Brain Res. (2014) 271:116–21. 10.1016/j.bbr.2014.05.068 24914461

[69] Alper K Schwartz KA Kolts RL Khan A. Seizure incidence in psychopharmacological clinical trials: an analysis of Food and Drug Administration (FDA) summary basis of approval reports. Biol Psychiatry. (2007) 62:345–54. 10.1016/j.biopsych.2006.09.023 17223086

[70] Bachetti T Di Zanni E Adamo A Rosamilia F Sechi MM Solla P. Beneficial effect of phenytoin and carbamazepine on GFAP gene expression and mutant GFAP folding in a cellular model of Alexander's disease. Front Pharmacol. (2021) 12:723218. 10.3389/fphar.2021.723218 34950024 PMC8688807

